# Case report: Vertical preparation protocol for veneers

**DOI:** 10.4317/jced.60223

**Published:** 2023-04-01

**Authors:** María Granell-Ruiz, Cristina Rech-Ortega, Begoña Oteiza-Galdón, Kheira Bouazza-Juanes

**Affiliations:** 1 Universidad Europea de Valencia. Faculty of Health Sciences. Department of Dentistry; 2 Clinical and Applied in Dental and Implant-Prosthetics Research Group. Universidad Europea de Valencia. Faculty of Health Sciences. Department of Dentistry

## Abstract

The biologically oriented preparation technique (BOPT) consists of a vertical preparation of the tooth that involves a reduction to zero of the emergence anatomy, the creation of a new finish area, and immediate temporization, so that the gingiva is supported by a suitable prosthetic restoration. To this effect, it is not the restoration that adapts to the gingiva, but the gingiva that adapts to the restoration. This technique not only allows the gingiva around the tooth to stabilise, but over time it will also facilitate the achievement of a predictable coronal migration of the gingival margin. The present paper describes the protocol from dental preparation to the cementation of the BOPT veneers, highlighting the differences with the established protocol for full coverage crowns and the protocol we have followed for many years for veneers with finish lines.

** Key words:**Veneers, vertical preparation, BOPT.

## Introduction

On the topic of achieving maximum aesthetics in the anterior sector using minimally invasive restorations, ceramic veneers cannot be ignored. There is an undoubtedly increasing demand for this type of treatment in our dental offices, and thanks to advancements in techniques and materials we can affirm that nowadays, due to their highly aesthetic appearance, proven biocompatibility, and long-term predictability, they have become a reliable type of restoration for aesthetic treatments in the anterior sector (1,2).

One of the main problems we have found over time in patients with this type of restorations is the occurrence of recessions. There appears to be a direct relationship between the presence of a finish line in the design of the dental preparation and the appearance of recessions over time. At the tooth-cement-restoration interface, over time the gingiva tends to migrate to an area where there is greater stability, which is generally at a more apical level (3).

Vertical preparation with rotary “gingitage” is a type of prosthetic preparation that has been in existence for many years. In their work, the Italian authors Di Febo, Carnevale, Trebbi, and BonFiglioli focused particularly on the healing of the periodontal complex after its preparation, together with the design of the gingival margin and the degree of precision obtained with vertical preparations (4). However, it was Dr Ignazio Loi who transformed this technique into nothing less than a philosophy, known as BOPT (Biologically Oriented Preparation Technique). It is a conventional, “feather-edge”, subgingival vertical preparation, where instead of a finish line there is a finish area. The laboratory technician, under the clinician’s instruction and using the information provided by the soft tissues, will place the prosthetic margin in the subgingival position at the most appropriate height, depending on each specific case and according to the controlled invasion of the sulcus, at maximum depth of 1 mm (5). The correct apico-coronal position of the finish area with respect to the free gingival margin should always be within the clinical gingival sulcus and never more than 1 mm deep, even if there is a deeper healthy sulcus (6). The biologically oriented preparation technique (BOPT) consists of the vertical preparation of the tooth, entailing a reduction to zero of its emergency anatomy together with the creation of a new finish area and immediate temporization, so that the gingiva is supported by a suitable prosthetic restoration, which is key to this technique. To this effect, it is not the restoration that adapts to the gingiva, but the gingiva that adapts to the restoration. This technique not only allows the gingiva around the tooth to stabilise, but over time it will also facilitate a predicTable coronal migration of the gingival margin (5). 

The present paper describes the protocol from dental preparation to cementation of the BOPT veneers, highlighting the differences with the established protocol for full coverage crowns and the protocol we have followed for many years for veneers with finish lines.

The BOPT technique involves a series of steps that must be followed, referred by Dr. Ignazio Loi as “key points of the technique” (5): vertical preparation (elimination of the anatomical cementoenamel junction, CEJ); “gingitage” of the sulcus; relining and adjustment of the provisional restoration (clot preservation); adaptation profile (new prosthetic CEJ); timing of the impression; and laboratory procedure (new adaptation profile). The diagnostic wax-up and mock-up are essential tools for communication and evaluation of the final aspect of the restoration between the patient and the clinician (2).

## Case Report

**Figure 1:**
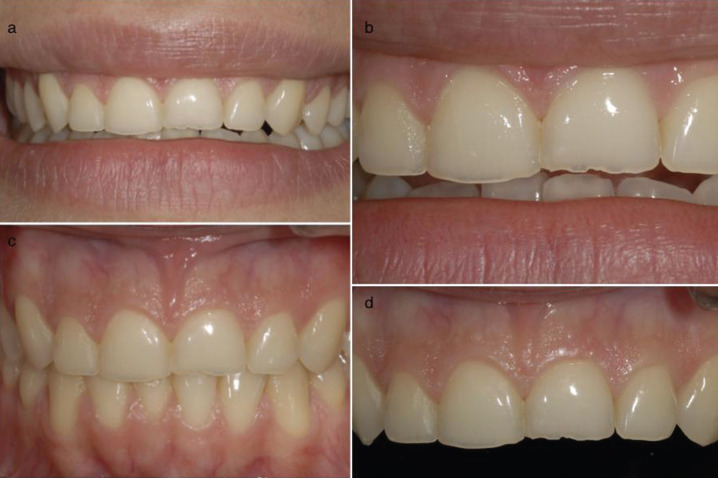
a-d) Initial clinical photos showing the wear on the incisal edges.

A 25-year-old female patient who came for consultation due to wear of the incisal edges of the four upper incisors due to the presence of a parafunctional habit. To improve both aesthetics and function, four ceramic veneers using the vertical preparation technique (BOPT) are proposed (Fig. 1).

1. Vertical preparation

Vertical preparations are characterised by the absence of a finish line in the dental preparation, eliminating the emerging anatomy of the tooth at cervical level. Before starting the preparation, a careful intrasulcular mapping around the entire tooth to assess the depth of the sulcus and the position of the bone crest must be performed, thereby carrying out a controlled invasion of the sulcus. Probing prior to the preparation is one of the basic pillars of the BOPT technique. If the CEJ cannot be located during the preparation, it will not be possible to erase or eliminate it, so rather than the gingival margin adapting to the provisional restoration during the temporization phase, it will gradually migrate apically until it finds the anatomical CEJ. According to Loi, the anatomic limit of the preparation must be the CEJ, so it is recommended that the bur does not come into contact with the crest. Once the CEJ is located, tooth preparation takes place (5). The burs used for vertical preparation are the #862 flame diamond burs with green, red, and yellow rings (Komet®, Intensiv®, Sweden & Martina®, etc.) 

**Figure 2:**
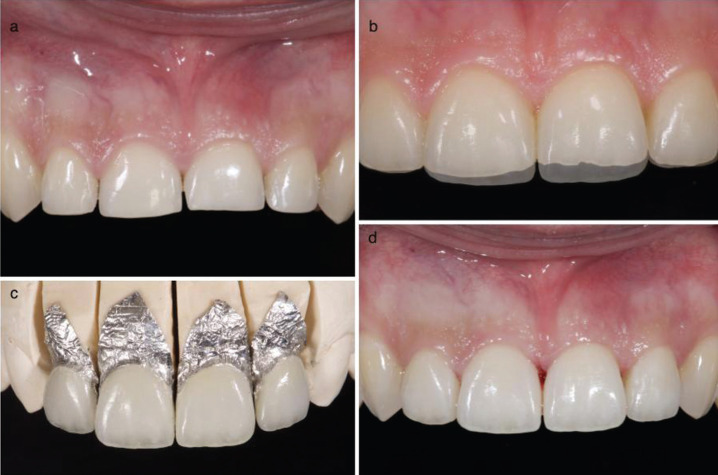
a) Dental preparation for BOPT-type veneers. b) Overlap of worn teeth and the future veneers. c) Veneers manufactured with the platinum foil technique. d) BOPT-type veneers immediately after cementation.

At this point, the difference between preparing a tooth to receive a full coverage crown or a veneer is simply the amount of the dental structure eliminated. For veneer preparations with finish lines, the #6844 bur is used (Komet®, Intensiv®, etc.), which is a slightly cone-shaped diamond bur with a rounded tip and gradual granulometry. A chamfer-type finish line is achieved using this bur, normally located at juxtagingival level. To prepare the tooth for a BOPT veneer, however, the green ring #862 flame diamond bur will be used first, with an intrasulcular inclination of 10-15º, and will be finished off using the same burs, but with a finer grain (ring colour red/yellow). In both cases, irrespective of whether the preparation includes a finish line or not, the contact points between the teeth must be broken with metal strips (Fig. 2a).

2. Gingitage of the sulcus

Dental preparation using the BOPT technique involves the simultaneous rotary curettage of the sulcus, which causes it to bleed and creates a space between the internal wall of the sulcus and the axial wall of the tooth. This space will fill with blood, which will later form a clot. This clot will be supported by the provisional restoration, producing a thickening of the gingival tissue that will surround the definitive restoration (7).

Rotary curettage is performed both in preparations for full coverage crowns and in preparations for veneers, although the latter is limited to vestibular level.

3. Relining and adjustment of the provisional restoration.

In preparations for full coverage crowns, the laboratory technician manufactures a “pre-preparation” provisional restoration that will be relined and adjusted. The function of this temporary restoration is to preserve the clot and ensure the correct maturation of the gingival tissues (5). In the case of veneers, resin provisional restorations are not requested in advance since they are required only for total coverage crowns. Instead, the temporary veneers are manufactured after the preparation by means of a “mock-up” based on the waxing performed previously by the technician. Once the mock-up has been put in place, it is recommended to penetrate the sulcus again using the #862 flame bur with a red or yellow ring, so that it bleeds again. This way the mock-up will preserve the clot and will allow the maturation of the gingival tissues until the definitive restorations are cemented (5).

In preparations for veneers with finish lines, the provisional restorations are also prepared from the mock-up and adjusted to the finish line of the preparations.

4. Adaptation profile

Loi *et al*. state that the gingival tissue will adapt to the shape of the tooth. It is the dental emergence profiles that determine the shape, scalloping and gingival thickness. The BOPT technique is innovative in that it creates an emergence profile that is not linked to the gingival profile, called an “adaptation profile” (8).

Due to the complete elimination of the emerging anatomy of the tooth during the preparation, the tissues are released from the support of the underlying hard tissues and will adapt to the provisional restorations from the mock-up. In other words, the gingiva will be able to read the shape and adapts to the provisional restorations. When a conventional finish line is prepared, there is no modification of the soft tissues, since the provisional restorations are limited by the finish line placed juxtagingivally (9).

5. Timing of the impression

In full coverage crowns, the minimum waiting time until the impression is four weeks, giving enough time for the clot to stabilise around the provisional restoration and become epithelial tissue, and for healing to occur until the tissues are properly mature and sTable (5).

In the case of BOPT veneers, the final impression will be taken on the same day the preparation is performed, using the double thread technique for gingival retraction. The patient uses the mock-up as the provisional restoration for the duration of the tissue healing period, which will coincide with the time it takes the laboratory technician to manufacture the definitive restorations (10). In preparations for veneers with a finish line, impressions are usually taken on the day the teeth are prepared.

6. Laboratory procedure

One of the key concepts of the BOPT technique is the creation of adaptation profiles capable of guaranteeing soft tissue stability, which cannot be obtained when conventional preparations are performed with a finish line. In this case, the laboratory technician manufactures the restorations adjusted to the finish line, making it more difficult to provide them with the ideal symmetry given by the vertical preparation (5.6). On the contrary, the lack of a finish line prepared by the clinician means the laboratory technician can determine a wider finish area where the ending of the prosthetic crown will be established and where the emergence profiles of the gingiva can be perfectly adapted to the coronal emergence profiles artificially created in the laboratory. The ending of both a prosthetic crown and a veneer is usually carried out at 0.5 mm from the gingival margin, with the laboratory technician providing the restorations with a new emergence profile and ideal proportions and symmetry, (Fig. 2b,c).

Once the restorations are in the dental office, the provisional restorations are removed and the final restorations are tested to verify that everything is as expected. They are then cemented using the conventional technique for cementing veneers. If the preparation is done with a finish line, the restorations must fit perfectly with the line (1,2).

After cementing the restorations, the gingiva should ideally bleed again so that the definitive restorations preserve the clot and the gingiva matures and stabilizes adjusted to the shape and emergence profile of the definitive restorations. The bleeding is produced passing a dental probe between the restoration and the gingiva. The patient is instructed not to brush their teeth for approximately 48 hours to preserve the clot, (Fig. 2d).

**Figure 3:**
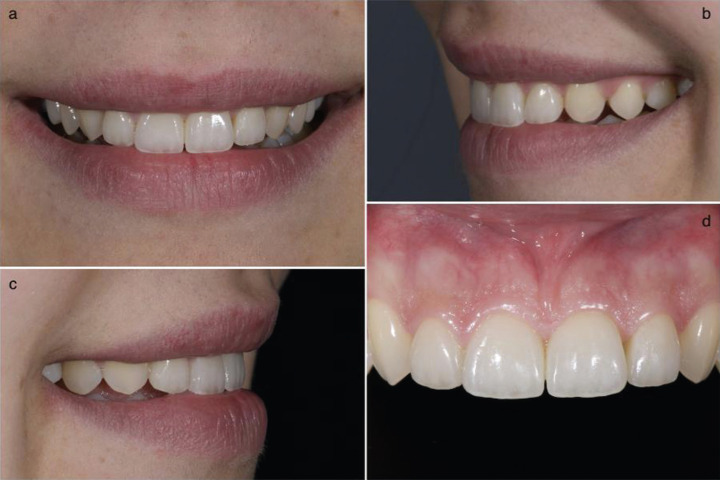
a-d) Final photos of BOPT-type veneers one month after cementation.

After a few months, the gingiva adapts to the shape established with the restorations, (Fig. 3).

## Discussion

Ceramic veneers are currently the aesthetic restorations par excellence in the field of dentistry. As has been verified over the years and tested in numerous clinical studies, this type of restoration has a sTable behaviour due to the intrinsic properties of the ceramic material itself (1,2).

Nonetheless, both the clinician and the patients themselves expect not only the restorations but also the surrounding tissues to remain sTable and free of any kind of inflammation that could lead to undesirable gingival recession over time. In this regard, dental preparation without a finish line could prevent what clinicians have observed for many years in this type of restorations when they are prepared with a finish line. The stability of gingival parabolas that has been observed in full coverage restorations prepared with the BOPT technique can be transferred to ceramic veneers (5).

With the BOPT technique, both the clinician and the laboratory technician can interact with the surrounding tissues, modifying its shape and architecture. The advantages are considerable given that most of the clinical results are obtained solely through the restoration itself, including both the temporary and the final restoration (margin position, emergent profile, tooth shape). More clinical and biological studies are needed to add scientific value to this technique (5).

As has been observed in various studies, gingival thickness increases with significant results. Soft tissue growth and increased vascularity appear to help achieve long-term stability (3,7,11,12).

The basis for correct aesthetics, function and comfort of the dentition is the periodontium and all prosthodontic treatments and restorative therapies require a healthy periodontium as a prerequisite for a successful outcome. The interplay between periodontal and restorative dentistry is present in many aspects, including the location of the restorative margins, the crown contours, and the response of the gingival tissues to the restorative preparations. Gingival recession may be directly involved in basic concepts of periodontal-restorative interactions, particularly with regard to interactions at the gingival margin of the restorations (3).

If dental preparations are not performed correctly, the fixed prostheses that sit on them can produce iatrogenic effects on the surrounding gingival tissues. Most research and meta-analyses of the published literature that analyse survival and the complications that may arise over the years focus more on the state of the restorations and place less emphasis on complications or problems at gingival or periodontal level. Just a handful of studies have investigated gingival health and how it behaves in treatments with fixed restorations, confirming that recession appears not only because of the age of the patient but also depends on the dental preparation carried out (13).

Likewise, other authors have observed that the presence of teeth with restorations with horizontal finish line preparations, compared to untreated teeth, can influence increases in the plaque index and the depth of probing, producing migration of the gingival margin and attachment loss (14,15).

## Conclusions

The BOPT technique currently represents a new trend in tooth preparation within the field of prosthetic dentistry. This paper demonstrates that this technique, which works when performed with full coverage restorations, can also work with ceramic veneers, allowing stability of the gingival tissues and avoiding unwanted recession.
